# Individual- and community-level factors associated with intimate partner controlling behavior among women in Somaliland: a multilevel analysis of the 2020 Somaliland demographic and health survey

**DOI:** 10.3389/fpubh.2026.1953781

**Published:** 2026-10-07

**Authors:** Abdulkadir Mohamed Nuh, Abdirashiid Muhumed Guled, Mohamed Abdilahi Mohamed, Abdisalam Hassan Muse, Hamse Arab Ali, Hamse Adam Abdi, Abdeta Muktar Ahmed, Omran Salih, Ridwan A. Mohamed

**Affiliations:** 1 School of Graduate Studies, University of Hargeisa, Hargeisa, Somalia; 2 School of Postgraduate Studies and Research, Amoud University, Borama, Somalia; 3 School of Graduate Studies and Research, Gollis University, Hargeisa, Somalia; 4 College of Medicine and Health Science, University of Hargeisa, Hargeisa, Somalia

**Keywords:** intimate partner controlling behavior, intimate partner violence, multilevel analysis, Somaliland, women

## Abstract

**Background:**

Intimate partner controlling behavior (IPCB) is a key manifestation of coercive control and an important precursor to other forms of intimate partner violence. Despite its adverse consequences for women’s autonomy, mental health, and well-being, evidence on the prevalence and factors associated with IPCB in Somaliland remains limited. This study aimed to estimate the prevalence and identify the individual- and community-level factors associated with IPCB among ever-married women in Somaliland.

**Methods:**

A secondary analysis of the 2020 Somaliland Demographic and Health Survey (SLDHS) was conducted using a weighted sample of 9,428.14 (unweighted *n* = 9,593) ever-married women aged 15–49 years. IPCB was defined as experiencing at least one of five partner controlling behaviors reported for the respondent’s current husband/partner. Multilevel logistic regression models, incorporating the DHS sample weight (V005) and clustering by primary sampling unit (135 clusters), were fitted to account for the hierarchical structure of the survey data and to identify factors associated with IPCB. Adjusted odds ratios (AORs) with 95% confidence intervals (CIs) were reported, and statistical significance was declared at *p* < 0.05.

**Results:**

The prevalence of IPCB was 26.7% (95% CI: 25.8–27.6%; unweighted prevalence 25.1%). Women aged 25–29 years (AOR = 1.60, 95% CI: 1.01–2.53), those with primary education (AOR = 1.70, 95% CI: 1.44–2.00), and women from middle- (AOR = 1.46, 95% CI: 1.20–1.77) and rich-wealth households (AOR = 1.56, 95% CI: 1.30–1.88) had higher odds of experiencing IPCB. Conversely, joint healthcare decision-making (AOR = 0.69, 95% CI: 0.60–0.79), current pregnancy (AOR = 0.73, 95% CI: 0.63–0.85), marriage at ages 19–26 years (AOR = 0.71, 95% CI: 0.63–0.80) or >26 years (AOR = 0.63, 95% CI: 0.49–0.81), household size of 4–7 members (AOR = 0.81, 95% CI: 0.71–0.92), partner employment (AOR = 0.77, 95% CI: 0.68–0.87), and residence in Marodijeh, Togdheer, Sool, and Sanaag regions were associated with lower odds of IPCB.

**Conclusion:**

Approximately one in four ever-married women in Somaliland experienced intimate partner controlling behavior, underscoring IPCB as an important public health and gender equality concern. Both individual- and community-level factors were significantly associated with women’s odds of experiencing IPCB, highlighting the need for multilevel interventions that strengthen women’s decision-making autonomy, delay early marriage, promote girls’ education, and implement context-specific strategies to prevent coercive control and advance gender equality.

## Introduction

Intimate partner controlling behavior (IPCB) is defined as a pattern of coercive tactics used by a partner to maintain dominance and restrict a woman’s personal agency (1). Conceptually, these behaviors such as monitoring movements and limiting social contact constitute a fundamental violation of human rights that undermines the physical and mental health of women (2).

The eradication of such control is central to the United Nations Sustainable Development Goal (SDG) 5, which aims to eliminate all forms of violence against women in both public and private spheres by 2030 (3). Furthermore, addressing IPCB is an essential objective of the African Union’s Agenda 2063, specifically under Aspiration 6, which envisions a people-driven Africa where gender equality is realized across all social and political domains (4). While earlier frameworks like the Millennium Development Goals (MDGs) focused on gender parity in education, current regional and global agendas recognize that the persistence of controlling behavior remains a significant obstacle to total female empowerment (5).

Statistical figures reveal that IPCB is a pervasive global crisis, with particularly high prevalence rates documented in sub-Saharan Africa. Recent multi-country analyses estimate that over half of women in several African nations report experiencing at least one form of partner control (6). Globally, the World Health Organization notes that while physical violence is widely tracked, controlling behavior often remains an underreported precursor that affects approximately one in three women in their lifetime (7). In East Africa, recent data suggest that jealousy and movement restrictions are experienced by nearly 43% of ever-partnered women (8). These figures highlight an urgent need for empirical research to unmask the hidden dynamics of domination within intimate relationships (9).

Historically, the study of domestic abuse was primarily confined to physical manifestations of violence, often neglecting the insidious nature of psychological and coercive tactics (10). However, the current study of intimate partner violence has evolved toward a “coercive control” framework, which views IPCB as a strategic method of entrapment (11). Modern research has firmly established that non-physical controlling behaviors are significantly associated with long-term psychological distress, including anxiety and depression (12). Recent evidence from low-income settings emphasizes that these behaviors often escalate into physical harm when women attempt to assert their independence (13). Contemporary scholarship also links IPCB to adverse reproductive health outcomes, such as reduced contraceptive use and increased risk of unintended pregnancy (14). Furthermore, current studies indicate that accusations of infidelity and restrictions on family contact are the most common forms of control reported in traditional societies (15). This historical shift in focus highlights that controlling behavior is not an isolated domestic issue but a systematic effort to undermine a woman’s humanhood (16).

The contextual background of Somaliland offers a unique environment for the study of IPCB due to its deeply patriarchal social structure and the influence of customary laws. In this region, traditional systems of authority often dictate gender roles, emphasizing female subservience and male dominance within the household (17). While Somaliland has made institutional progress, cultural norms surrounding “honor” often dictate that domestic matters, including partner control, remain private (18). The 2020 Somaliland Demographic and Health Survey (SLDHS) provides the first robust opportunity to examine these dynamics in Somaliland since it captures comprehensive data on women’s experiences of autonomy and violence (19). Given the conservative social fabric, it is essential to understand how traditional expectations of “submissiveness” intersect with modern socioeconomic changes to influence the prevalence of partner control (20).

The identification of factors associated with IPCB requires a careful examination of various individual and community-level covariates. At the individual level, studies have consistently found that younger women and those with lower educational attainment are significantly more vulnerable to partner control (21). Marital status and the duration of the union also play a role, with those in early years of marriage often facing higher levels of surveillance (22). The dual nature of economic empowerment is also a critical covariate; while a woman’s employment can enhance her agency, it may also trigger controlling behaviors from partners who feel their traditional authority is being challenged (23). Community-level factors such as the region of residence, household wealth index, and rurality have also been identified as significant predictors of IPCB (24). Furthermore, frequency of media exposure and ownership of a personal bank account are increasingly recognized as factors that either mitigate or exacerbate the risk of partner domination (3). Finally, the normalization of violence within a community acts as a potent covariate that sustains the acceptability of controlling tactics (25).

Despite the wealth of data provided by the 2020 SLDHS, a critical gap exists in the literature regarding a specific and isolated focus on IPCB in Somaliland. Most existing research aggregates controlling behavior into broader categories of emotional violence, thereby failing to capture its distinct predictors and its role as a precursor to physical abuse (26). Furthermore, there is a lack of studies utilizing multilevel modeling to account for the hierarchical structure of the data, where individual experiences are nested within community clusters (27). This study’s novelty lies in its application of a multilevel logistic regression approach to disentangle individual-level factors from community-level influences in the Somaliland context (28). By focusing specifically on Somaliland, this research provides a culturally tailored analysis that moves beyond generalized sub-Saharan African trends (29).

The primary purpose of this study is to assess the prevalence and identify the multi-level factors associated with intimate partner controlling behavior among reproductive-age women in Somaliland. By utilizing the 2020 SLDHS data, this research contributes to a deeper understanding of how personal characteristics and community environments interact to shape women’s autonomy (30). The findings are intended to inform the design of evidence-based interventions and policies aimed at reducing gender-based violence and promoting gender equality in Somaliland (30). Ultimately, this work seeks to empower women by highlighting the factors that sustain partner domination and advocating for social changes that align with global human rights standards (31).

## Methods

### Study area

The study was conducted in Somaliland, in the Horn of Africa, using the six primary regions defined in the 2020 Somaliland Health and Demographic Survey (SLHDS): Awdal, Maroodi Jeex, Sahil, Togdheer, Sool, and Sanaag. These regions include both urban and rural populations, although rural and pastoral communities constitute a substantial proportion of the population. Urban settlement is concentrated mainly in major centers such as Hargeisa, Borama, Berbera, Burao, Las Anod, and Erigavo. The regions also vary in access to healthcare, education, and other social services, providing an important socio-cultural context for examining intimate partner dynamics and controlling behaviors.

### Study design and data source

This study employed a cross-sectional study design based on a secondary analysis of the 2020 SLDHS. The 2020 SLDHS is a nationally representative survey designed to provide data on a wide range of monitoring and evaluation indicators in the areas of population, health, and nutrition. For the purpose of this research, the dataset was filtered to include only respondents from the Somaliland regions. The survey utilized a multi-stage cluster sampling technique to ensure that the findings are representative of the target population at both the regional and national levels.

### Population and sample

The study population consisted of ever-married women of reproductive age (15–49 years) residing in the selected households in Somaliland at the time of the survey. A total of 9,593 women with complete data on all study variables were included in the analytic sample (unweighted n).

The analysis used the individual sampling weight (V005) provided in the SLDHS dataset. The original V005 sampling weight was retained and used for the survey-weighted analysis. Among the 9,593 retained respondents, the sum of the V005 weights was 9,428.14.

Consistent with the SLDHS sampling frame, the survey covered urban and rural settlements only; the nomadic population was not included in the 2020 SLDHS. Consequently, the findings of this study may not be generalizable to nomadic women in Somaliland, who may face different social and structural exposures relevant to IPCB.

### Sample weighting

To account for the complex survey design, the primary sampling unit (cluster, V001; 135 clusters represented in the analytic sample) was specified as the level-2 grouping unit in all multilevel models. Sampling strata were constructed by crossing region (V024) with type of place of residence (V025), yielding 12 strata, as the public-release SLDHS file does not include a separate stratum identifier. The complex design was declared using svyset, and the rescaled sample weight was applied as an importance weight (iw = wt) in both the descriptive statistics and the multilevel logistic regression models.

### Study variables

The primary outcome variable of this study was IPCB, treated as a binary categorical variable (experienced vs. not experienced). This variable was constructed from five specific indicators, each referring to the respondent’s current husband/partner: whether he (a) is jealous if the respondent talks to other men, (b) accuses her of unfaithfulness, (c) prohibits her from meeting female friends, (d) limits her contact with family, or (e) insists on knowing her location at all times. Each item was originally coded as Yes, No, or Do not know; “do not know” responses were treated as missing prior to constructing the outcome. If a woman reported at least one of these five behaviors, she was categorized as having experienced IPCB (coded 1); women reporting “no” to all five items were coded 0. Because these items ask about the current partner’s behavior, the resulting measure reflects current/recent controlling behavior rather than lifetime experience across all partners.

Media access was assessed based on respondents’ access to three sources of information: television, radio, and newspapers. Respondents who had access to at least one of these media sources were categorized as “Yes,” whereas those who had no access to any of the three sources were categorized as “No.”

Partner employment status was coded as a binary variable, with respondents whose partners were employed categorized as “Yes” and those whose partners were not employed categorized as “No.”

### Data analysis

The data analysis was performed using Stata version 17. Descriptive statistics and multilevel logistic regression to account for the hierarchical nature of the SLDHS data, where individuals are nested within communities (clusters). Descriptive statistics, including weighted frequencies and percentages, were used to summarize the characteristics of the sample and the prevalence of IPCB. For the inferential analysis, four models were developed: Model 0 (the null model) to estimate community-level variance without predictors; Model I, containing only individual-level variables; Model II, containing only community-level variables; and Model III (the full model), incorporating both levels. The Intraclass Correlation Coefficient (ICC) was calculated to determine the proportion of variance attributable to community-level differences. Model fitness was evaluated using the Log Likelihood, Akaike Information Criterion (AIC), and Bayesian Information Criterion (BIC), with the lowest AIC indicating the best-fitting model. Statistical significance was determined at *p*-values of less than 0.05, 0.01, and 0.001.

Model III (the full model) was selected as the final model based on its lowest AIC; AIC was prioritized over BIC because BIC imposes a heavier penalty on cluster-level parameters in multilevel models, which can favor an overly parsimonious specification when the research objective is to jointly estimate individual- and community-level factors. Likelihood-ratio tests comparing nested models were additionally conducted to formally test model improvement, using twice the difference in log-likelihood between nested models (see Results, Multilevel Model Comparison).

The Median Odds Ratio (MOR) and Proportional Change in Variance (PCV) were computed for each model to provide a more interpretable measure of the contextual [cluster-level) heterogeneity underlying the ICC; MOR was calculated as exp. (0.6745 × √(2σ^2^)] and PCV as [(σ^2^Model 0 − σ^2^Model x)/σ^2^Model 0] × 100, with the computed values reported for each model in Table 1 and described in the Multilevel Model Comparison subsection of the Results.

**Table 1:** Weighted frequency and percentage distribution of sample characteristics *N* = 9,428.

Variable	Categorical	Weighted frequency	Percentage
Intimate partner controlling behavior	No controlling behavior	6,907.90	73.3
	Experienced controlling behavior	2,520.20	26.7
Maternal age	15–19 years	129.6	1.4
	20–24 years	673.4	7.1
	25–29 years	1,803.20	19.1
	30–34 years	2,075.90	22
	35–49 years	4,746.10	50.3
Sex of household head	Male	5,733.90	60.8
	Female	3,694.20	39.2
Maternal education	No education	7,709.70	81.8
	Primary	1,353.50	14.4
	Secondary/Higher	364.9	3.9
Total children ever born	Less than five	1,764.80	18.7
	Five or more	7,663.30	81.3
Decision on healthcare	Respondent	2,570.10	27.3
	Jointly	3,951.00	41.9
	Husband	2,907.00	30.8
Wealth status	Poor	3,649.80	38.7
	Middle	985.9	10.5
	Rich	4,792.40	50.8
Currently pregnant	No pregnancy	8,145.40	86.4
	Pregnancy	1,282.70	13.6
Age at first marriage	12–18 years	5,266.70	55.9
	19–26 years	3,736.70	39.6
	Over 26 years	424.7	4.5
Household size	1–3 members	1,904.70	20.2
	4–7 members	5,477.30	58.1
	8 or more members	2,046.10	21.7
Media access	No media access	1,818.30	19.3
	Has media access	7,609.80	80.7
Partner worked in last 12 months	No	4,532.40	48.1
	Yes	4,895.70	51.9
Smoking cigarette	No	9,390.40	99.6
	Yes	37.8	0.4
Owns a house	No	4,521.80	48
	Yes	4,906.30	52
Owns land	No	8,081.90	85.7
	Yes	1,346.20	14.3
Has bank account	No	9,232.40	97.9
	Yes	195.8	2.1
Region	Awdal	805.4	8.5
	Marodijeh	2,940.10	31.2
	Sahil	604.2	6.4
	Togdheer	2,579.40	27.4
	Sool	1,018.10	10.8
	Sanaag	1,480.90	15.7
Place of residence	Rural	5,567.30	59.1
	Urban	3,860.80	40.9

Given that IPCB prevalence exceeded 10%, we considered estimating adjusted prevalence ratios as an alternative to odds ratios, since odds ratios can overstate the corresponding relative risk for outcomes of this frequency. We elected to retain odds ratios as the primary effect measure for consistency with the comparator multi-country DHS literature cited throughout this manuscript, which similarly reports adjusted odds ratios from multilevel logistic models; this choice is noted as a limitation, and prevalence ratios are identified as a useful direction for future work.

### Ethical considerations

The study relied on a secondary analysis of de-identified data from the 2020 SLDHS. The original survey was conducted according to international ethical guidelines, with informed consent obtained from all participants before the interviews. The DHS Program granted the researchers permission to use the dataset for this specific study. Because the data were completely anonymized and contained no personal identifiers, this study was exempt from further institutional review board approval. The researchers maintained the confidentiality and integrity of the data throughout the analysis process, ensuring that the findings are reported accurately and ethically.

## Results

A total of 9,593 ever-married women with complete data were included in the analysis (weighted *n* = 9,428.14). The multilevel models used 135 primary sampling units (clusters) as the level-2 grouping unit.

### Sample characteristics

The weighted study sample comprised 9,428 women, with the majority residing in rural areas (59.1%). More than half were married at ages 12–18 (55.9%), and about half were aged 35–49 years (50.3%). Educational attainment was low, with 81.8% having no formal education, while 81.3% had five or more children. Regarding household characteristics, 60.8% lived in male-headed households, and joint healthcare decision-making was reported by 41.9% of respondents. Overall, 26.7% of women experienced at least one form of intimate partner controlling behavior, whereas 73.3% reported no controlling behavior (Table 2).

**Table 2:** Descriptive statistics for intimate partner controlling behavior (IPCB) (weighted).

Intimate partner controlling behavior	Frequency	Percentage
Husband jealous if respondent talks with other men		
No	7,799.57	82.73
Yes	1,628.57	17.27
Husband accuses respondent of unfaithfulness		
No	8,511.42	90.28
Yes	916.72	9.72
Husband does not permit respondent to meet female friends		
No	8,189.67	86.86
Yes	1,238.47	13.14
Husband tries to limit respondent contact with the family		
No	8,727.45	92.57
Yes	700.68	7.43
Husband insists on knowing where respondent is		
No	8,245.78	87.46
Yes	1,182.36	12.54

### Prevalence of intimate partner controlling behavior

The Figure 1 shows that 73.3% of women reported no intimate partner controlling behavior, while 26.7% experienced at least one form of controlling behavior. This indicates that approximately one in four women reported experiencing controlling behavior from an intimate partner.

**Figure 1:**
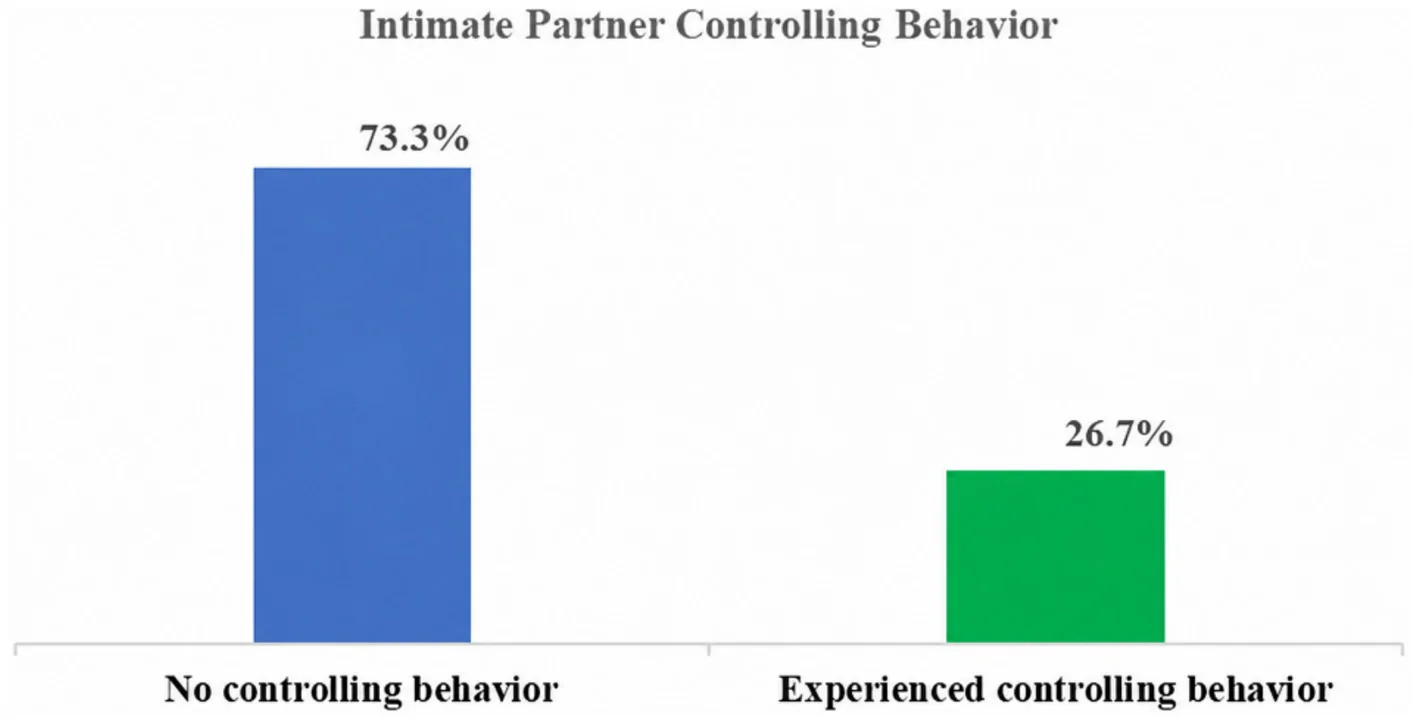
Distribution of intimate partner controlling behavior among women in Somaliland.

### Description of intimate partner controlling behavior in each component

As shown in Figure 2 and Table 3, the weighted descriptive statistics for intimate partner controlling behavior indicate that the most prevalent form of control experienced by respondents is partner jealousy when they speak to other men, reported by 17.27% of the sample. This is followed by restrictions on social and physical autonomy, where 13.14% of respondents are prevented from seeing female friends and 12.54% report that their husbands insist on knowing their exact location at all times.

**Table 3:** Multilevel logistic regression odds ratios for intimate partner controlling behavior (IPCB).

Predictor	Model 0 (Empty)	Model I	Model II	Model III
Maternal age	Ref: <20 years			
20–24 years		1.46 [0.91, 2.32]		1.43 [0.89, 2.28]
25–29 years		1.65 [1.05, 2.61] *		1.60 [1.01, 2.53] *
30–34 years		1.60 [1.01, 2.55] *		1.51 [0.95, 2.42]
35–49 years		1.67 [1.05, 2.67] *		1.58 [0.99, 2.53]
Sex of household head	Ref: Male			
Female		1.12 [1.00, 1.24] *		1.11 [1.00, 1.24]
Maternal education	Ref: No education			
Primary		1.67 [1.43, 1.97] ***		1.70 [1.44, 2.00] ***
Secondary higher		0.86 [0.61, 1.21]		0.86 [0.61, 1.20]
Total children	Ref: <5 children			
Five and more		1.02 [0.87, 1.20]		1.05 [0.89, 1.23]
Decision healthcare	Ref: Alone			
Jointly		0.69 [0.60, 0.79] ***	0.68 [0.59, 0.77] ***	0.69 [0.60, 0.79] ***
Husband		1.27 [1.11, 1.46] **	1.20 [1.05, 1.37] **	1.28 [1.11, 1.46] **
Wealth status	Ref: Poor			
Middle		1.50 [1.24, 1.81] ***	1.35 [1.11, 1.63] **	1.46 [1.20, 1.77] ***
Rich		1.60 [1.33, 1.91] ***	1.43 [1.21, 1.71] ***	1.56 [1.30, 1.88] ***
Currently pregnant	Ref: No			
Pregnancy		0.75 [0.64, 0.87] ***		0.73 [0.63, 0.85] ***
Age at first marriage	Ref: <19 years			
19–26 years		0.73 [0.65, 0.82] ***		0.71 [0.63, 0.80] ***
Over 26 years		0.64 [0.50, 0.82] ***		0.63 [0.49, 0.81] ***
Household size	Ref: <4 members			
4–7 Members		0.80 [0.71, 0.92] **		0.81 [0.71, 0.92] **
8 + members		0.99 [0.85, 1.16]		0.99 [0.85, 1.17]
Media access	Ref: No			
Has media access		0.89 [0.78, 1.01]	0.96 [0.84, 1.10]	0.92 [0.81, 1.06]
Partner works	Ref: No			
Yes		0.83 [0.74, 0.94] **		0.77 [0.68, 0.87] ***
Smokes cigarette	Ref: No			
Yes		0.67 [0.34, 1.34]		0.64 [0.32, 1.27]
House owner	Ref: No			
Yes		1.05 [0.94, 1.18]		1.08 [0.97, 1.21]
Land owner	Ref: No			
Yes		1.16 [1.00, 1.34] *		1.13 [0.97, 1.31]
Bank account				
Yes		0.94 [0.65, 1.37]		0.96 [0.65, 1.40]
Region	Ref: Awdal			
Marodijeh			0.40 [0.30, 0.54] ***	0.35 [0.26, 0.48] ***
Sahil			0.90 [0.70, 1.16]	0.83 [0.64, 1.08]
Togdheer			0.52 [0.39, 0.69] ***	0.45 [0.34, 0.60] ***
Sool			0.70 [0.55, 0.89] **	0.60 [0.47, 0.78] ***
Sanaag			0.53 [0.41, 0.69] ***	0.44 [0.33, 0.57] ***
Residence	Ref: Rural			
Urban			1.05 [0.92, 1.20]	1.00 [0.88, 1.15]

* *p* < 0.05, ** *p* < 0.01, *** *p* < 0.001; Ref, reference category.

**Figure 2:**
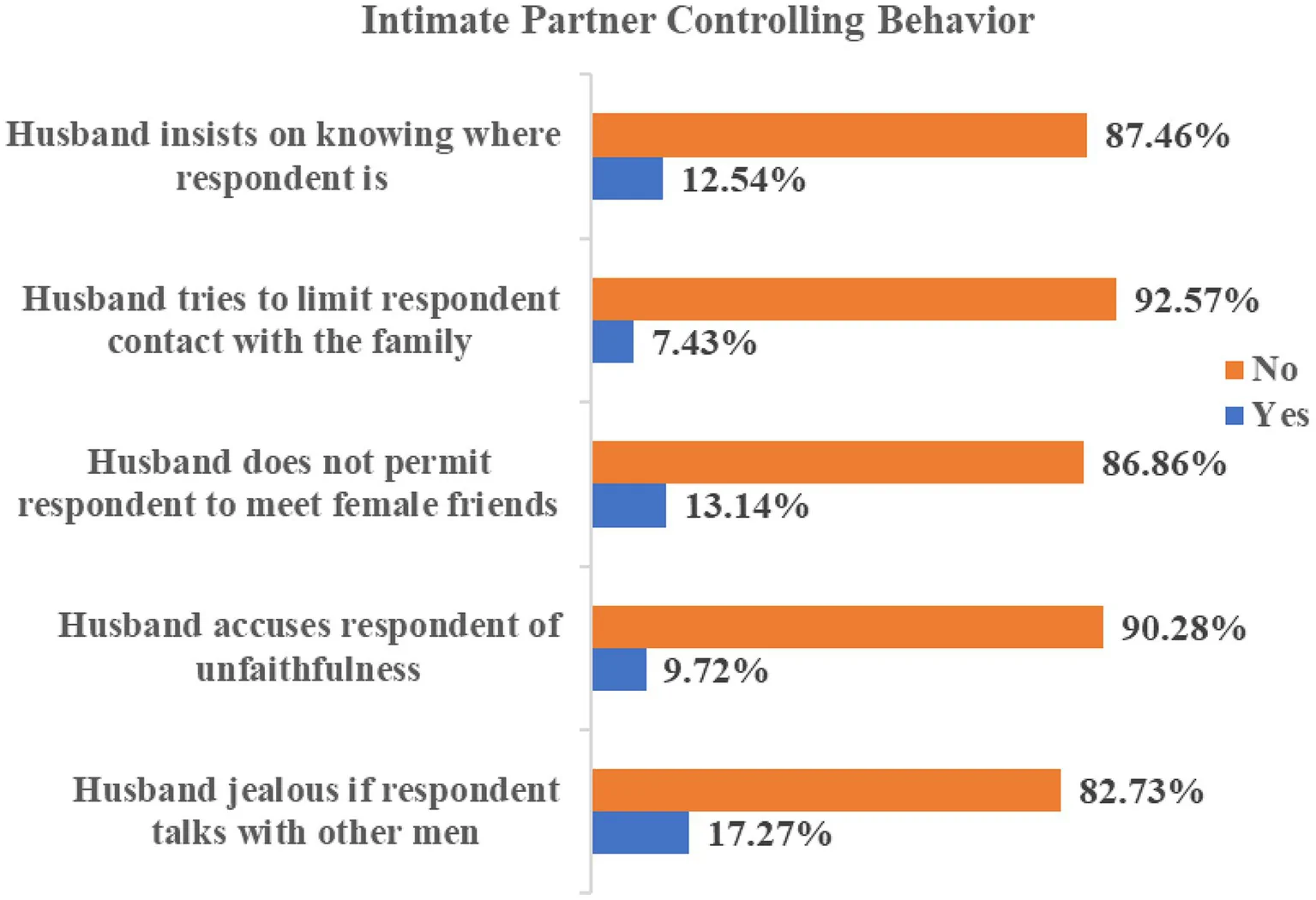
Description of intimate partner controlling behavior in each component.

Accusations of unfaithfulness are reported by 9.72% of the participants, while the least common behavior involves attempts to limit the respondent’s contact with their own family, which occurs in 7.43% of cases. Although the majority of the population ranging from 82.73 to 92.57% across the various indicators reports an absence of these specific behaviors, the data shows that nearly one in five women faces emotional or social monitoring, with isolation from friends and surveillance of location being the primary methods of control after jealousy.

### Individual- and community-level factors associated with IPCB

The multilevel logistic regression analysis identifies several significant individual and community-level predictors of IPCB.

In the final model (Model III), women aged 25–29 had 60% higher odds of experiencing IPCB compared to those under age 20, and women with primary education had 70% higher odds than those with no education, though higher education levels did not show a significant effect. Socioeconomic status was a strong predictor, with women in middle (AOR = 1.46) and rich (AOR = 1.56) wealth categories facing significantly higher odds of IPCB than those in the poor category.

Healthcare decision-making dynamics were also significant; joint decision-making was associated with 31% lower odds of IPCB, whereas the husband making healthcare decisions alone was associated with 28% higher odds of IPCB. Other factors associated with lower odds of IPCB included being currently pregnant (AOR = 0.73), marrying at an older age specifically over 26 years (AOR = 0.63) or between 19 and 26 years (AOR = 0.71) and having a partner who was employed in the last 12 months (AOR = 0.77).

Household size was also associated with IPCB, as women in households with 4 to 7 members had lower odds of experiencing IPCB compared to those in smaller households. Geographically, compared to the Awdal region, women living in Marodijeh, Togdheer, Sool, and Sanaag had significantly lower odds of experiencing controlling behavior, with Marodijeh showing the lowest odds (AOR = 0.35). Notably, variables such as urban versus rural residence, ownership of a bank account, and media access did not show statistically significant associations with IPCB in the full model (Table 4).

**Table 4:** Multilevel model comparison: Random-Effect Variance, ICC, MOR, PCV, and Model Fit Statistics.

Parameter	Model 0	Model I	Model II	Model III
Number of women	9,593	9,593	9,593	9,593
Number of clusters (V001)	135	135	135	135
Cluster variance, σ^2^ (SE)	1.84 (0.32)	1.72 (0.31)	1.71 (0.31)	1.81 (0.32)
ICC (%)	35.9	34.3	34.2	35.5
MOR	3.65	3.49	3.48	3.61
PCV (%)	Reference	6.5	7.1	1.6
Log likelihood	−5026.71	−4902.13	−4936.63	−4861.59
AIC	10057.41	9854.26	9899.26	9785.17
BIC	10071.75	10033.48	9992.46	10007.40

ICC, intra-class correlation coefficient; MOR, median odds ratio; PCV, proportional change in variance; AIC, Akaike information criterion; BIC, Bayesian information criterion.

ICC was calculated as σ^2^/(σ^2^ + 3.29). MOR was calculated as exp. [0.6745 × √(2 × σ^2^)]. PCV was calculated as [(Variance in Model 0 − Variance in subsequent model)/Variance in Model 0] × 100.

### Multilevel model comparison

The model comparison table evaluates the performance and fit of four nested multilevel logistic regression models used to analyze the factors associated with IPCB. The random effects analysis shows a cluster variance ranging from 1.71 to 1.84, which indicates significant variation in the outcome at the community level. The Intraclass Correlation Coefficient (ICC) ranges from 34.2 to 35.9%, suggesting that approximately one-third of the total variance in IPCB is attributable to differences between communities or clusters, rather than individual differences alone. In terms of model fit, the Log Likelihood increases from −5026.71 in the empty model to −4861.59 in the full model (Model III), indicating that the addition of both individual and community-level variables improves the model’s fit to the data.

Likelihood-ratio tests, calculated as twice the difference in log-likelihood between nested models, confirmed that each successive model provided a statistically significant improvement in fit relative to the null model: Model 0 vs. Model I, χ^2^ = 249.16; Model 0 vs. Model II, χ^2^ = 180.16; and relative to the full model, Model I vs. Model III, χ^2^ = 81.08, and Model II vs. Model II, χ^2^ = 150.08 (all *p* < 0.001), supporting the inclusion of both individual- and community-level predictors.

The number of clusters (135 primary sampling units) was consistent across all four models. The Median Odds Ratio (MOR) and Proportional Change in Variance (PCV) were computed for each model to provide a more interpretable measure of contextual heterogeneity: MOR ranged from 3.48 (Model II) to 3.65 (Model 0), indicating substantial cluster-level heterogeneity in IPCB even after adjustment for individual- and community-level covariates (Model III MOR = 3.61), while PCV showed that individual-level factors accounted for 6.5% of the baseline cluster variance and community-level factors for 7.1%, with the full model explaining only 1.6% relative to the null model together indicating that most of the between-cluster variation in IPCB remains unexplained by the measured covariates.

This improvement is confirmed by the Akaike Information Criterion (AIC), which reaches its lowest value in Model III (9785.17), identifying the full model as the best fit for the data.

Although the Bayesian Information Criterion (BIC) shows its lowest value in Model II (9992.46), the full model (Model III) was retained as the primary model given the study’s objective of jointly estimating individual- and community-level factors, and because AIC is generally preferred over BIC in multilevel models given BIC’s stronger penalty on cluster-level parameters.

## Discussion

The prevalence of IPCB observed in Somaliland (26.7%) is comparable to findings reported in several sub-Saharan African settings. A recent multilevel analysis of Demographic and Health Survey (DHS) data from eight sub-Saharan African countries reported country-specific IPCB prevalence ranging from 3.68 to 27.56%, with the upper estimate closely approximating the prevalence observed in the present study (1). Similarly, a pooled analysis of 19 sub-Saharan African countries found that 28.9% of women experienced high levels of partner controlling behavior (32). Although the 2016 Ethiopia Demographic and Health Survey assessed intimate partner violence rather than controlling behavior specifically, it reported a prevalence of 28.7%, suggesting a comparable burden of partner abuse within the region (33).

Considerably higher levels of partner controlling behavior have been documented in several sub-Saharan African studies, with more than half of women experiencing at least one controlling behavior (5). Conversely, lower prevalence estimates have also been reported, with country-specific figures as low as 3.68% in the recent multi-country DHS analysis (1). These variations are likely attributable to differences in the study design, sampling strategies, sociocultural norms, women’s empowerment, and the effectiveness of gender equality and violence prevention interventions. Nevertheless, the present finding indicates that IPCB remains a significant public health and human rights concern in Somaliland.

Women aged 25–29 years had higher odds of experiencing IPCB than women younger than 20 years. Although several studies have reported greater vulnerability among younger women, age-related associations are not uniform across settings. Multicountry evidence from sub-Saharan Africa indicates that women’s age is an important correlate of partner violence and controlling behavior, but that the direction and magnitude vary by relationship duration, economic dependence and prevailing gender norms (1, 2, 10). The higher odds among women aged 25–29 years in Somaliland may reflect increasing marital responsibilities, childbearing and negotiations over household resources during the early and middle stages of marriage.

Women with primary education had higher odds of IPCB than women with no formal education. This non-linear pattern differs from the general evidence that higher educational attainment protects women against intimate partner violence (IPV). A meta-analysis of 44 DHS datasets from 29 sub-Saharan African countries found that women with less than secondary education had a significantly greater risk of IPV than those with secondary or higher education (34). IPCB-specific evidence from South Africa has similarly linked lower educational attainment to a higher likelihood of partner-controlling behavior specifically, rather than physical or sexual IPV alone (2, 3). Education may improve awareness of rights and increase women’s willingness to challenge restrictions.

Household decision-making was significantly associated with IPCB. Compared with women who made healthcare decisions alone, women who made such decisions jointly with their partners had lower odds of IPCB. This finding is consistent with multicountry evidence showing that women’s participation in household decisions is associated with a lower likelihood of IPV (35). Similar associations have been reported in Rwanda (36), Ethiopia (37) and wider sub-Saharan Africa (35), where limited involvement in decisions concerning healthcare, household purchases and family visits was linked to greater partner violence. This pattern has also been documented specifically for controlling behavior, rather than IPV broadly, in South African survey data (2, 3). Joint decision-making may indicate communication, mutual respect and a more equitable distribution of power. This proposed mechanism reflects a plausible hypothesis rather than a directly tested pathway, as communication quality and relationship dynamics were not independently measured in this survey.

Unexpectedly, women from middle- and high-wealth households had higher odds of IPCB than women from poor households. This contrasts with much of the IPV literature, which identifies poverty and economic dependence as important risk factors (34, 38). However, evidence specifically examining controlling behavior suggests that household wealth may operate differently from its relationship with physical or sexual violence. A South African study identified household wealth as a significant determinant of IPCB, while broader evidence indicates that economic change can provoke controlling responses when women’s access to resources challenges established gender hierarchies (2, 39). In wealthier Somaliland households, partners may have more assets, communication devices and social relationships to monitor or restrict. The result may also reflect greater recognition or disclosure of subtle controlling acts among economically advantaged women. Because household wealth does not necessarily measure women’s personal control over resources, economic prosperity at household level should not be interpreted as equivalent to women’s financial autonomy.

Marriage at an older age was strongly associated with lower odds of IPCB. Women who married at 19–26 years and those who married after 26 years had lower odds of IPCB than women married before age 19. This is consistent with evidence linking early marriage to greater vulnerability to partner violence (40, 41). Girls who marry early frequently enter unions with limited education, financial resources, negotiating capacity and social support, often alongside a substantial spousal age and power imbalance. Delaying marriage may therefore be associated with lower IPCB by allowing women additional time to complete education, develop social networks and acquire greater psychological and economic autonomy before entering marriage. The finding reinforces the importance of enforcing minimum-age-at-marriage legislation and expanding educational and livelihood opportunities for adolescent girls.

Women living in households of four to seven members had lower odds of IPCB than those in households with fewer than four members. Moderate household size may offer women greater social presence, emotional support or informal oversight that constrains overt partner control. However, very large households may provide no additional protection because of crowding, economic pressure or hierarchical family relations (42). These proposed mechanisms are plausible hypotheses rather than directly tested pathways, as this study did not include validated measures of social support, communication quality, or women’s empowerment. As evidence concerning household composition and IPCB remains limited, this association should be investigated further using information on household relationships and co-residence with extended family members.

Partner employment was also associated with lower odds of IPCB: women whose partners were working had 23% lower odds of IPCB. This accords with evidence connecting unemployment and economic insecurity with IPV and controlling behavior (43). Employment may reduce financial stress and interpersonal conflict while strengthening men’s socially expected provider role. Conversely, unemployed partners may use monitoring, jealousy and restrictions as alternative means of asserting authority when economic roles are threatened. Nevertheless, the quality and stability of employment, rather than employment status alone, may determine whether work is associated with lower or higher partner control.

Marked geographical differences were also observed. Compared with Awdal, the odds of IPCB were substantially lower in Marodijeh, Togdheer, Sool and Sanaag. Geographic heterogeneity in partner violence has been documented throughout sub-Saharan Africa, reflecting variations in gender norms, conflict exposure, socioeconomic development, service access and willingness to disclose abuse (43). The particularly high relative burden in Awdal cannot be explained by the measured individual characteristics alone and may indicate contextual influences such as locally specific marital norms, community acceptance of male authority, economic conditions or differences in access to protection and support services. Regionally disaggregated qualitative and spatial research is therefore required before attributing these differences to any single cultural or structural factor.

The findings of this study showed that intimate partner controlling behavior remains an important concern among women in Somaliland, with approximately one in four women reporting at least one form of controlling behavior. The observed associations with socioeconomic, household, and decision-making characteristics suggest that IPCB is shaped not only by individual factors but also by broader gender and power dynamics within households. These findings highlight the need for interventions that strengthen women’s autonomy, promote equitable household decision-making, address harmful gender norms, and improve access to social and economic support. Future research should use longitudinal and qualitative approaches to better understand the pathways linking socioeconomic conditions, household power relations, and controlling behavior. From a policy perspective, prevention efforts should be integrated into broader women’s empowerment, reproductive health, and gender-based violence programs in Somaliland.

## Conclusion

Approximately one in four ever-married women in Somaliland experienced intimate partner controlling behavior, underscoring IPCB as an important public health and gender equality concern. Both individual- and community-level factors were significantly associated with women’s odds of experiencing IPCB, highlighting the need for multilevel interventions that strengthen women’s decision-making autonomy, delay early marriage, promote girls’ education, expand women’s economic opportunities, integrate IPCB screening and support within reproductive and primary healthcare services, engage men and community leaders in addressing harmful gender norms, and strengthen referral pathways for women experiencing coercive control.

### Limitations

This study has several limitations that should be considered when interpreting the findings. First, the 2020 SLDHS sampling frame covered urban and rural settlements only and did not include the nomadic population; findings may therefore not generalize to nomadic women in Somaliland. Second, the cross-sectional design of the SLDHS precludes causal inference; associations reported here should be interpreted as correlational, and reverse causality cannot be ruled out for example, IPCB could plausibly influence subsequent household decision-making patterns rather than the reverse. Third, IPCB was measured through self-report, which may be subject to social desirability bias and under-reporting given the sensitivity of the topic and cultural norms around disclosure of domestic matters.

Fourth, several covariates, including household wealth, capture household-level resources rather than women’s individual economic autonomy and should not be interpreted as measures of the latter. Fifth, two covariates smoking status (0.4% of the weighted sample) and bank account ownership (2.1% of the weighted sample) had very small proportions in the “yes” category, which may produce unstable coefficient estimates; findings for these two predictors should be interpreted with caution. Sixth, effect estimates are reported as odds ratios rather than prevalence ratios, and IPCB was analyzed using a single threshold (≥1 of 5 behaviors) rather than a graded severity measure; both were retained for consistency with the comparator DHS literature cited throughout, and represent useful directions for future sensitivity analyses. Finally, unmeasured constructs such as social support, communication quality, and women’s empowerment could not be directly assessed with the available SLDHS variables and represent important directions for future qualitative and mixed-methods research.

## Data Availability

The raw data supporting the conclusions of this article will be made available by the authors, without undue reservation.
